# The Ottawa Charter in practice: community-led action to reduce health inequalities in the UK

**DOI:** 10.1093/heapro/daag105

**Published:** 2026-08-03

**Authors:** Lisa McNally

**Affiliations:** Department of Applied Health Sciences, University of Birmingham, Edgbaston, Birmingham B15 2TT, United Kingdom; Worcestershire County Council, Wildwood, Wildwood Drive, Worcester WR5 2QT, United Kingdom

**Keywords:** health promotion, Ottawa Charter, community development, health inequalities, place-based, co-production

## Abstract

This article presents a practice-based case study illustrating how the action areas and strategies outlined in the Ottawa Charter can be put into practice and reduces neighbourhood-level health inequalities. The Priority Neighbourhood Development (PND) programme in Worcestershire focused on small, high-need geographical areas identified through analyses of emergency hospital admissions and socio-economic data. Work in the socio-economically deprived Westlands housing area is described in detail. Community development workers mapped local assets and deficits, enabling residents to challenge stigmatizing narratives and build positive place identity. A resident-led working group was established and supported with a devolved budget, facilitating locally determined actions such as expanding counselling provision, enhancing community facilities, developing arts and nature-based projects, and improving skills through training and youth-led initiatives. Co-location and outreach by health, housing, voluntary sector, and policing partners reoriented services towards prevention and accessibility. Evaluation after 18 months showed reductions in emergency hospital admissions and children’s social care referrals, improved wellbeing, and reduced social isolation. Qualitative feedback highlighted increased empowerment, strengthened community action, and improved access to support. The model has since been scaled across the County and embedded into local policy and commissioning frameworks. The PND Programme demonstrates how neighbourhood-level investment, resident leadership, and cross-sector mediation can meaningfully reduce health inequalities and build healthier, more resilient communities.

Contribution to Health Promotion

- This article describes an application of the Ottawa Charter for Health Promotion in public health practice in the UK.
- The Worcestershire Priority Neighbourhood Programme is a community-led initiative that transfers resources and power to people living and working in socio-economically deprived neighbourhoods.
- The work is described using a framework based on the action areas and strategies proposed within the Ottawa Charter.

## Introduction

In recent decades, health promotion in England has focused on changing individuals’ lifestyles. However, despite significant investment in this work, health inequalities across England remain persistent (Office for National Statistics 2026).

The Ottawa Charter (WHO 1986) served to expand the concept of health promotion. It highlights that health is shaped by a complex web of interconnected factors and not influenced solely by individual behaviours (Thomas *et al*. 2025). The Charter proposes five key action areas: public policy, creating supportive environments, strengthening community action, developing personal skills, and reorientating health services. Strategies for achieving this action include advocacy, mediation across agencies and communities, and enabling the reduction of health inequality.

While the Ottawa Charter proposes a focus on the ‘place’ as well as the ‘individual’, there has been little agreement in subsequent practice about what ‘place’ actually means. Some studies have applied The Charter to national level action (Taylor *et al*. 2009). Others have focused on smaller institutions such as schools (Gugglberger 2021). An English study highlighted the greater utility of focusing on smaller geographical units (Hood *et al*. 2021). Specifically, this research analysed lower super output areas (LSOAs), containing populations of 1000 to 3000 residents, when examining the determinants of need and service demand.

This article provides a case study of an English programme that aims to enable place-based, community-led change in some of the most economically deprived lower super output areas. The Worcestershire Priority Neighbourhood Development (PND) programme was developed and evaluated according to key Ottawa Charter actions and strategies. Led by the County’s Public Health Department, PND devolved power and budgets to local people, fulfilling the ambition set out by Shiroya *et al*. (2026) of ‘Putting Civil Society in the driving seat’. It also aimed to reorientate services provided by healthcare and local authorities, a Charter action area that has often been overlooked (Ziglio *et al*. 2011).

## Materials and methods

### Programme implementation

**Figure 1:**
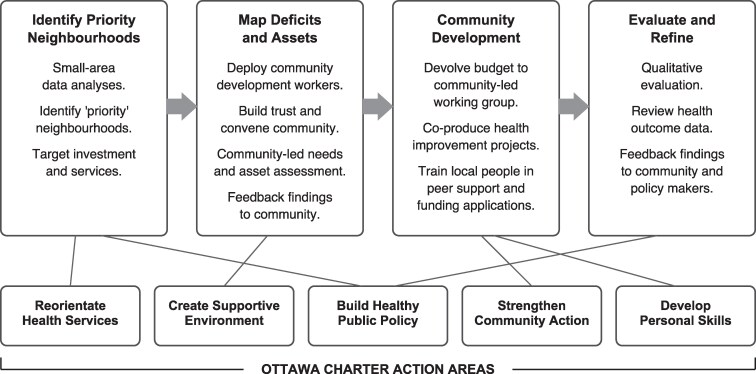
Stages of the PND programme and links to Ottawa Charter action areas.

The PND Programme implemented a four-stage model of community-led health promotion work: (i) identify high-need neighbourhoods; (ii) map assets and deficits; (iii) community development; and (iv) evaluate and refine. The model is illustrated in Fig. 1 along with how the stages relate to the five action areas proposed by the Ottawa Charter. Pilot work evaluated implementation and impact in one deprived LSOA: the Westlands Housing Estate in Droitwich.

#### Identification and advocacy for priority neighbourhoods

To identify high-need neighbourhoods, statistical process control analyses were applied to LSOA level data on emergency hospital admissions and socio-economic factors. This was used to identify neighbourhoods with emergency hospital admission rates more than three standard deviations above the mean. Unplanned, emergency visits to hospital were understood to be a useful indicator of where high levels of health need were not matched by adequate health promotion or healthcare provision. The Westlands Housing Estate was chosen for piloting the next stages of the PND process due to its particularly high level of deprivation.

#### Mapping of assets and deficits

Community development workers were deployed to the Westlands Housing Estate to build up a profile of the strengths and weaknesses in the area. These workers were employed within the local public health team. They were trained in the facilitation of community action, enabling local residents to identify their needs and initiate new projects. These workers also served as a vital link between communities and statutory health and care organizations.

Needs assessment prior to the community development stage took the form of unstructured interviews with local people living and working in the Westlands area. These focused not only on need, but also community strengths, including local assets, community projects, and service provision. The interviews were captured and edited into a short film co-produced with residents called ‘We Are Westlands’ (Worcestershire County Council 2023). The findings of this needs assessment are reported in the Results section of this report.

#### Community development

Stage three in the PND process followed the key Ottawa Charter strategies of advocacy, enabling and mediation. A working group of local people was established to direct the work and enabled with resources and support. This group was made up of Westlands residents, voluntary workers and teachers at the local school. Links were also formed between the working group and local healthcare providers. A budget of £100,000, funded by the local Public Health Department, was devolved to the working group through a grant. The group then created a plan for how the budget would be spent and how services and resources should be engaged.

Meetings were facilitated between local community members and the commissioners and providers of statutory organizations. These included healthcare services and the local authority. These meetings focused on advocacy for service reorientation and enhanced provision of services in the area.

#### Qualitative evaluation

The evaluation of the programme began around 18 months after its launch. The aim was to examine the impact of the programme and how well it had adhered to the five Ottawa Charter action areas.

Information on community-led activities, along with qualitative feedback on the programme from local people, was gathered and recorded by staff from the local public health team. Feedback from local people was given during unstructured interviews during the planning and co-production of a second film: ‘We are Westlands Now!’ (Worcestershire County Council 2025). Information from the review of activities and feedback was then collated by the author into key themes based on the Charter’s five action areas.

Written consent was gained from participants for their responses to be used and disseminated through online publication of both films. Consent was also gained for the use of their quoted responses in this report.

#### Quantitative evaluation

Quantitative analyses were conducted of service demand data at a small-area level. These included urgent hospital admissions and children’s social care data. Data was analysed at two time points; before programme implementation and at 18 month follow up. Changes in the outcomes within the Westlands LSOA were compared with the average changes across the rest of Worcestershire where the PND programme had not been implemented.

## Ethical approval

This article described a practice case study of a programme delivered as part of the Worcestershire County Council statutory responsibilities to improve public health. No individual quantitative data is reported. Quotes from local people are confined to those who gave written consent to appear in the promotional film and for inclusion of their quotes in this research report. Ethical approval has been granted from the University of Birmingham Humanities and Social Sciences Research Ethics Committee (Ethical Review: ERN_5805-Mar2026).

## Results

### Needs assessment

Prior to the implementation of the community development stage, local people identified a range of needs and deficits. These included poverty, poor mental health, domestic abuse, a lack of support to parents, and social isolation. Poor education facilities and provision of health services were also cited by local people, although the staff working in those services were highly regarded.

Many strengths and assets were also highlighted, including positive and influential individuals, a strong sense of community and the local Wellbeing Hub, which provided counselling and practical help with health and social issues. However, the Hub was struggling to find resources and was in danger of having to close.

### Action area #1: creating supportive environments

At follow up, a review of community-led activity delivered within the programme highlighted that the funding had been used to improve the Wellbeing Hub. This resulted in an expansion of its capacity to provide support, and the reconstruction of rooms for community activities. Support has also been enhanced by the establishment of a new support group, led by a local parent, for families of children with special educational needs. Sustainability was also evident, in that the working group had successfully applied for further funding for the Wellbeing Hub from additional, external charitable sources.

### Action area #2: strengthening community action

A key theme emerging from the feedback was that local people felt empowered by the way in which the community were entrusted with resources and enabled to lead the programme. One resident said: ‘*The main thing is that we’re listened to, and I think that’s brilliant*’.

A wide range of community actions resulted from the programme. For example, projects to improve the environment were initiated, such as the establishment of a nature trail around the housing area’s limited green spaces. Benches were installed along the trail at the request of working group members who lived with poor physical mobility. These benches were installed by a local tradesman from the housing estate.

Some of the funding was used to establish a youth-led bicycle repair project, which enhanced skills and self-confidence. This also had the effect of allowing local people on low incomes access to working bicycles, increasing opportunities for physical activity.

### Action area #3: developing personal skills

The local school received support to deliver a variety of arts projects around the area, including the creation of a mural designed by children. The funding was also used to create a new reading pod in the school. A school staff member reported that ‘*The community grant enabled us to give the children the very best, which is what I believe they deserve. Having a brand-new reading pod, which is lovely and a really nice environment. The children can use that to read and enjoy. And also we can involve other members of the community*’.

The central involvement of the local school, alongside the extra funding sourced for training, demonstrated a clear focus on developing skills and providing more opportunities for local people. Training in employability skills, IT and computing, sports coaching, counselling, and food hygiene allowed adults and children in Westlands to create their own futures. In addition, residents involved in the working group developed and enhanced a range of personal skills such as negotiation and resource management.

### Action area #4: reorienting health services towards prevention and equity

Through mediation between residents and statutory services, local service provision was increased in the Westlands housing area, including access to health checks and advice on long term condition self-management. Healthcare service outreach was enhanced in the area, supplemented by the provision of a range of physical and mental health improvement programmes.

At follow up, interviews with local stakeholders revealed themes around improved health, mental wellbeing and social isolation. Workers and volunteers at the hub were able to deliver a 136% increase in counselling hours. They also reported that several people had told them that they would have ended their lives if it was not for the support they received there.

The analysis of area level service data revealed a year-on-year reduction in emergency hospital admissions of −4.9%, which compared well to Countywide data showing an increase in admissions of +6.8% (an overall difference of 11.7%). Children’s social care referrals also decreased in Westlands by −12.7%, compared with a countywide reduction of −3.7% (a difference of 9%).

### Action area #5: building healthy public policy

The implementation and evaluation of the pilot work in Westlands led to significant changes to local health and care policy. The PND programme has been adopted as a central health and care strategy in Worcestershire. Additional, recurrent funding of over a million pounds per year has been secured for PND work across the County and it has informed the development of Worcestershire’s response to the UK Government’s Neighbourhood Health Programme.

## Discussion

The specific activities delivered in Westlands were shaped by local circumstances, but the PND model has several core features that are intended to be transferable. These include small-area intelligence to identify priority neighbourhoods, community development capacity to build trust and engagement, resident-led decision-making supported by devolved resources, mediation between communities and statutory services, and iterative evaluation. By contrast, the precise neighbourhood selected, the local partners involved, and the projects funded are context-specific and should be determined by local needs, assets and relationships.

While the results were deliberately analysed in a way that aligned with the five action areas of the Ottawa Charter, some activity could be seen as relating to more than one action area. For example, creating a supportive environment in the Wellbeing Hub also brought people together and strengthened community action. This strengthening of community action, in turn, led to meetings with health service staff that reorientated those health services. As Fry and Zask (2017) highlight, the fact that the Ottawa Charter’s action areas are not silos is one of the Charter’s strengths. They interact and depend on each other in mutually reinforcing ways.

The Charter’s ambition of strengthening community action was achieved in Westlands by devolving power and financial resources to local people. Resident control of funds is a step-change from consultation to shared governance, directly aligning with enablement and strengthening community action. This approach has been integrated into countywide healthcare commissioning policy and PND is being implemented across several other areas with an allocation of significant and sustained financial resources. This aligns well with the ambition set out by Shiroya *et al*. (2026) who, in relation to the Ottawa Charter, observed that ‘*We will know we are on course when community action is not just a title but a line item in budgets*’.

The PND programme was not originally designed as a research study. As such, limitations are inherent in the evaluation. In particular, the attribution of the positive changes in service demand to the programme cannot be made with full confidence. However, it is notable that similar positive changes were not seen in the comparative countywide data, and qualitative work did reveal themes consistent with improvements in health and wellbeing.

## Conclusion

The PND Programme provides a case study illustrating the value of small-area investment and community-led development in line with the Ottawa Charter. It created supportive environments, strengthened community action, developed personal skills, and reorientated health services. The result was improved wellbeing and reduced health and social care demand.

PND had a significant influence on healthy public policy across the rest of Worcestershire. It is also influencing national practice after it was recognized with the national LGC Public Health Award in 2025.

## Data Availability

Not applicable.
